# OP7, a novel influenza A virus defective interfering particle: production, purification, and animal experiments demonstrating antiviral potential

**DOI:** 10.1007/s00253-020-11029-5

**Published:** 2020-12-04

**Authors:** Marc D. Hein, Heike Kollmus, Pavel Marichal-Gallardo, Sebastian Püttker, Dirk Benndorf, Yvonne Genzel, Klaus Schughart, Sascha Y. Kupke, Udo Reichl

**Affiliations:** 1 Bioprocess Engineering, Otto von Guericke University Magdeburg, Magdeburg, Germany; 2 Department of Infection Genetics, Helmholtz Centre for Infection Research, Braunschweig, Germany; 3 Bioprocess Engineering, Max Planck Institute for Dynamics of Complex Technical Systems, Magdeburg, Germany; 4 University of Veterinary Medicine Hannover, Hannover, Germany; 5 Department of Microbiology, Immunology and Biochemistry, University of Tennessee Health Science Center, Memphis, Tennessee USA

**Keywords:** Influenza A virus, Antiviral, Defective interfering particles, Cell culture-based production, Steric exclusion chromatography, Animal experiments, OP7

## Abstract

**Abstract:**

The novel influenza A virus (IAV) defective interfering particle “OP7” inhibits IAV replication in a co-infection and was previously suggested as a promising antiviral agent. Here, we report a batch-mode cell culture-based production process for OP7. In the present study, a seed virus containing standard virus (STV) and OP7 was used. The yield of OP7 strongly depended on the production multiplicity of infection. To inactivate infectious STV in the OP7 material, which may cause harm in a potential application, UV irradiation was used. The efficacy of OP7 in this material was preserved, as shown by an *in vitro* interference assay. Next, steric exclusion chromatography was used to purify and to concentrate (~ 13-fold) the UV-treated material. Finally, administration of produced OP7 material in mice did not show any toxic effects. Furthermore, all mice infected with a lethal dose of IAV survived the infection upon OP7 co-treatment. Thus, the feasibility of a production workflow for OP7 and its potential for antiviral treatment was demonstrated.

**Key points:**

*• OP7 efficacy strongly depended on the multiplicity of infection used for production*

*• Purification by steric exclusion chromatography increased OP7 efficacy*

*• OP7-treated mice were protected against a lethal infection with IAV*

**Supplementary Information:**

The online version contains supplementary material available at 10.1007/s00253-020-11029-5.

## Introduction

With annual epidemics and occasionally severe pandemics, influenza A virus (IAV, list of abbreviations, Table 1) is a major human pathogen. Every year, about 300,000–650,000 deaths are reported worldwide (Iuliano et al. 2018). The current countermeasures include the use of vaccines and antivirals like oseltamivir and zanamivir (Colman 2009; Oxford 2007; Smith et al. 2006). However, the development and production of vaccines is a time-consuming process. Moreover, resistances arising against current antivirals have been reported and are a general issue (Han et al. 2018; Lackenby et al. 2018), demonstrating the need for novel treatment modalities.

**Table 1:** List of abbreviations

Abbreviation	Meaning
DCS	Differential centrifugal sedimentation
DI	Defective interfering
DIP	Defective interfering particle
FFU/mL	Focus forming units per mL
FL	Full length
GMEM	Glasgow minimal essential medium
HA	Hemagglutinin
hpi	Hours post infection
IAV	Influenza A virus
M1 protein	Matrix protein 1
M1-OP7	Mutated matrix protein 1
MDCK	Madin-Darby canine kidney
MOI	Multiplicity of infection
MS	Mass spectrometry
NC	Negative control
OP7	Overproportional segment 7
PBS	Phosphate-buffered saline
PFU/mL	Plaque forming units per mL
PR8	Influenza virus strain A/PR/8/34
PR8-RKI	PR8 provided by the Robert Koch Institute Germany
PR8-NIBSC	PR8 provided by the National Institute for Biological Standards and Control
real-time RT-qPCR	Real-time reverse transcription-qPCR
RT	Reverse transcription
SCMA	Sulfated cellulose membrane adsorbers
SEC	Size exclusion chromatography
Seg1–8	Segment 1–8
Segment-specific RT-PCR	Segment-specific reverse transcription-PCR
SPF	Specific pathogen free
STV	Standard virus
SXC	Steric exclusion chromatography
TCID_50_	50% tissue culture infective dose
VCC	Viable cell concentration
vRNA	Viral RNA

An approach that was previously suggested is the use of defective interfering (DI) particles (DIPs) as an antiviral agent (Alnaji and Brooke 2020; Dimmock et al. 2008; Scott et al. 2011a, b; Smith et al. 2016; Zhao et al. 2018). DIPs are naturally occurring virus mutants that have been observed for most DNA and RNA viruses. IAV DIPs typically contain a large internal deletion in one of their eight viral RNAs (vRNAs) (Davis et al. 1980). Thus, DIPs miss the genetic information required for the synthesis of a full-length protein essential for viral replication (Huang and Baltimore 1970). Therefore, DIPs are only capable of replication upon co-infection with an infectious standard virus (STV), compensating for the missing or truncated protein. Yet, in such a co-infection scenario, STV replication is inhibited and suppressed, and mainly non-infectious DIPs are released (Frensing et al. 2013; Frensing et al. 2014; Tapia et al. 2019; Von Magnus 1951). One explanation for this interference is the shorter DI vRNA, which may amplify faster compared with the full-length vRNA. Thus, the DI vRNA outcompetes STV replication for limited cellular or viral resources (Dimmock and Easton 2014; Laske et al. 2016; Nayak et al. 1985), which is here referred to as replication interference. In addition, it was suggested that DIPs can also interfere with virus replication unspecifically, via the enhanced induction of innate immunity (Easton et al. 2011).

Previously, an antiviral effect was shown upon DIP treatment in mouse and ferret models (Dimmock et al. 2012; Dimmock et al. 2008; Scott et al. 2011a; Smith et al. 2016). More recent results also showed an antiviral effect in mice experiments through a plasmid-based treatment, which encoded three IAV DI genes (Zhao et al. 2018). The interfering effect was directed against the replication of a diversity of IAV subtypes (Dimmock et al. 2008). Moreover, antiviral effects even against influenza B and pneumovirus replication was shown, likely mediated by the unspecific stimulation of innate immunity, conferred by DIP infection (Dimmock and Easton 2015; Easton et al. 2011; Scott et al. 2011a). The interference of IAV DIPs with a variety of IAV subtypes might indicate that, in contrast to vaccines, no annual adaptation to the currently circulating virus strain would be necessary (Dimmock and Easton 2015). An additional advantage of DIPs over vaccines is the faster mode of action. More specifically, vaccines induce an adaptive immune response; therefore, it can take several weeks for full protection. In contrast, DIPs interfere with the replication of the pathogen and therefore act immediately.

Recently, we discovered a novel type of DIP, termed OP7, in influenza virus strain A/PR/8/34 (PR8) (Kupke et al. 2019). Instead of a large internal deletion, OP7 contains 37 point mutations in segment 7 (Seg7) vRNA in relation to the reference sequence (from the National Center for Biotechnology Information). These point mutations affect the promoter regions, encoded proteins, and the packaging signal sequence. The OP7 phenotype displays an over-proportional level of Seg7 vRNA upon co-infection with STV, intracellularly and in the released virus particle population. Moreover, OP7 virions appear to miss a large proportion of other vRNA segments, which may explain its defect in virus replication. Interestingly, we observed a previously described “superpromotor” (Belicha-Villanueva et al. 2012) on Seg7 vRNA of OP7, which might explain the replication interference of OP7. Here, the predominant replication and transcription of Seg7 OP7 may take away resources, required for STV replication, leading to an enhanced propagation of OP7 over STV in a co-infection.

OP7 showed strong interference with the replication of IAV in Madin-Darby canine kidney (MDCK) cells and in human cell lines, as shown in *in vitro* co-infection experiments. This interference was not only directed against a common laboratory strain (i.e., PR8). We also demonstrated interference with very recent and relevant epidemic and pandemic human IAV strains, which were suggested by the WHO as vaccine strains (until ~2016–2018) (Kupke et al. 2019). Finally, the unspecific stimulation of innate immunity, observed upon OP7 co-infection, might even further promote the antiviral efficacy. Therefore, OP7 appears to be a very promising candidate for antiviral therapy.

In the present study, a small-scale cell culture–based process for the production and purification of highly effective OP7 material was established. For production, the interplay of DIPs and STV was assessed by testing a range of multiplicities of infection (MOIs) in order to obtain high OP7 yields. Next, the produced OP7 material was treated with UV irradiation, to ensure complete STV inactivation while maintaining a high interfering efficacy, as shown in an *in vitro* assay. Subsequently, the material was purified and concentrated using membrane-based steric exclusion chromatography (SXC). Lastly, animal experiments in a mouse model demonstrated the anti-IAV activity of OP7.

## Materials and methods

### Cells and viruses

An MDCK cell line (ECACC, No. 84121903) adapted to suspension growth (Lohr et al. 2010) was further adapted to grow in Xeno™ medium as described earlier (Bissinger et al. 2019). In this study, the chemically defined medium was used, instead of the serum free version. MDCK cells were cultivated either in (i) shake flask with 50-mL working volume (125-mL baffled polycarbonate Erlenmeyer Flask, Thermo Fisher Scientific, 4116-0125) at 37 °C, 5% CO_2_ and 185 rpm (Multitron Pro, Infors HT; 50-mm shaking orbit) or (ii) a STR with a working volume of 500 mL (DASGIP® Parallel Bioreactor System, Eppendorf AG, 76DG04CCBB) at 37 °C, ≥ 40% O_2_, pH 7.6, and 150 rpm (marine impeller). Viable cell concentration (VCC), cell viability, and cell diameter were determined using an automated cell counter (Vi-CELL XR, Beckman Coulter, 731050). Adherent MDCK cells from ECACC (No. 84121903) were cultivated in Glasgow minimum essential medium (GMEM) containing 1% peptone and 10% fetal bovine serum (cultivation medium) at 37 °C and 5% CO_2_.

Seed virus titers were determined by a 50% tissue culture infective dose (TCID_50_) assay using adherent MDCK cells (Genzel and Reichl 2007). In Table 2, an overview of all seed viruses used in this study is shown. All experiments with infectious virus were performed in laboratories with biological safety level 2 certification and ABSL animal facilities, following the respective safety regulations.

**Table 2:** Overview of seed viruses

Name	Purpose	Source virus	TCID_50_/mL	Seg7 OP7 vRNA copies/mL	Reference
OP7 seed virus	OP7 production (suspension cells)	PR8-NIBSC	1.3E+8	5.60E+10	Kupke et al. (2019)
STV	Control infection (suspension cells)	PR8-RKI	5.6E+8	–	Bissinger et al. (2019)
STV	Interference assay (adherent cells)	PR8-RKI	1.1E+9	–	
Plaque-purified STV	MS analysis	PR8-NIBSC	5.8E+8	–	Kupke et al. (2019)
Active OP7	Animal experiments	PR8-NIBSC	0 (UV-inactivated)	2.20E+10	This work
STV	Animal experiments	PR8 (plasmid based)	1.2E+9 (1.2E+8 FFU/mL)	–	Lambertz et al. (2018)

### Batch-mode production of OP7

The OP7 seed virus used for production (1.3E+8 TCID_50_/mL, 5.60E+10 Seg7 OP7 vRNA copies/mL) was derived from a single-cell virus isolate (“OP7-2”) as described previously (Kupke et al. 2019). The virus strain used in these single-cell experiments was IAV PR8 provided by the National Institute for Biological Standards and Control (PR8-NIBSC, No. 99/716). The seed virus for pure STV infections (5.6E+8 TCID_50_/mL) was adapted to growth in suspension MDCK cells as described previously (Bissinger et al. 2019) and originated from IAV strain PR8 provided by the Robert Koch Institute Germany (PR8-RKI).

For production, suspension MDCK cells were centrifuged (300×*g*, 5 min, room temperature). The complete medium was replaced with fresh, chemically defined Xeno™ medium. VCC was adjusted to 2E+6 cells/mL and trypsin added to a final concentration of 20 U/mL. MOIs were calculated based on TCID_50_ titers. Infected cell cultures were incubated at 37 °C, 5% CO_2_, and 185 rpm. Samples or final products were centrifuged (3000×*g*, 10 min, 4 °C) and stored at –80 °C until measurement.

### Interference assay

To evaluate the interfering efficacy of the produced OP7 material, an interference assay was used (adapted from (Kupke et al. 2019)). Here, the OP7-induced reduction in virus titers in a co-infection with STV was determined. Further, OP7 material was added with a fixed volume to allow the identification of production conditions yielding the highest interfering efficacy per product volume. First, 6-well plates were seeded with adherent MDCK cells (1E+6 cells per well) and incubated for 24 h. Before infection, cells were washed twice with phosphate-buffered saline (PBS). Then, cells were co-infected with infectious STV virus (see Table 2) at a MOI of 10 (based on TCID_50_ measurements) and 125 μL of produced OP7 material. Infection medium (for adherent cells, GMEM containing 1% peptone and 5 U/mL trypsin) was filled up to a total volume of 250 μL. After incubation (37 °C, 5% CO_2_, 1 h), cells were washed with PBS once. Then, 2 mL infection medium per well was added, and cells were incubated again (37 °C, 5% CO_2_, 16 h). Supernatants were analyzed for the hemagglutinin (HA) titer, plaque titer, and vRNAs in the progeny virions. The obtained HA and plaque titers were then used for a qualitative comparison.

### Virus quantification assays

The total number of IAV particles was quantified with a hemagglutination assay (Kalbfuss et al. 2008). For quantification of infectious IAV particles, either a TCID_50_ assay or a plaque assay was used. TCID_50_ (Genzel and Reichl 2007) measurements were used to determine the infectious titer of seed viruses and the infectious titer of samples of the production process. Plaque assay measurements were used for evaluation of the interference assay and for the inactivation kinetics after UV treatment.

For the plaque assay, 6-well plates were seeded with adherent MDCK cells (0.8E+6 cells per well) and incubated (37°C, 5% CO_2_, 48 h). Before infection, cells were washed twice with PBS, and the samples were prepared in serial 10-fold dilutions in infection medium. Of each dilution, 250 μL/well were incubated (37 °C, 5% CO_2_, 1 h). Afterward, the supernatant was removed, and the cells were overlaid with 1% agar in infection medium. Incubation (37°C, 5% CO_2_) was conducted for 4 days. The cells were fixed using methanol and stained with a 0.2% crystal violet solution. After fixation, the plaque count was determined by light microscopy and expressed as plaque forming units per mL (PFU/mL).

### PCR measurements

PCR-based approaches were used to analyze genomic vRNAs of virus particles. The vRNA of samples (supernatant of infected cells) was purified using the NucleoSpin® RNA virus kit (Macherey-Nagel, 740956) according to the manufacturer’s instructions. Segment-specific reverse transcription-PCR (segment-specific RT-PCR) was used to identify vRNAs containing a deletion (indicating DI vRNAs) for each IAV genome segment. Real-time reverse transcription qPCR (real-time RT-qPCR) was used to quantify vRNAs of IAV segment 5 (Seg5), segment 8 (Seg8) and the mutated Seg7 of OP7 (Seg7 OP7).

#### Segment-specific RT-PCR

Reverse transcription (RT) of isolated vRNAs and segment-specific PCR amplification of the resulting cDNAs were performed as described previously (Frensing et al. 2013; Kupke et al. 2019). Briefly, RT was performed in a single reaction using a universal primer, which hybridizes to the conserved 3′ region of all eight genome segments (Hoffmann et al. 2001). For the subsequent PCR, individual primers were used for each genome segment. PCR products were visualized using gel electrophoresis and investigated for the presence of short PCR products, indicating deleted DI vRNAs.

#### Real-time RT-qPCR

For the real-time RT-qPCR, we used a primer system of a previously reported method (Kawakami et al. 2011) that allows for gene-specific detection of individual IAV vRNA segments. *In vitro* generation of the reference standards, RT, real-time qPCR, and the calculation for absolute quantification of vRNA levels were conducted as described previously (Kupke et al. 2019). For the quantification of the mutated Seg7 OP7, we designed a new primer combination and generated a new reference standard. For this, the S7 OP7 vRNA sequence (GenBank accession number: MH085234) was synthesized and cloned into a pMX vector, conducted by GeneArt (Thermo Fisher). Next, the primers used for reference standard generation were “S7-OP7 Uni for” (AGTAAAAACAGGTAGATGTTGAAAG) and “S7-OP7 Uni T7 rev” (TAATACGACTCACTATAGGG AGTAGAAACAAGGTAGTTTTTTAC). The tagged primers used for RT are shown in supplementary table S1. Primers used for the real-time qPCR are shown in supplementary table S2.

### Matrix protein 1 analysis

Mass spectrometry (MS) was used to determine the abundance of mutated and total Matrix protein 1 (M1 protein), encoded by Seg7 vRNA, in the produced OP7 material. Before MS measurements, samples were heat inactivated (3 min, 80 °C).

#### Plaque purified STV generation as reference material

As a negative control (NC), a plaque-purified STV devoid of OP7 (derived from PR8-NIBSC virus), containing only the wild-type M1 protein, was used (as described in Kupke et al. 2019). Here, plaques from PR8-NIBSC viruses were picked and reseeded in three consecutive assays. Obtained viruses were then multiplied on adherent MDCK cells for the generation of a seed virus. In the present study, the seed virus was further propagated in adherent MDCK cells (MOI 1E−4, 24 hpi) to generate a larger stock (see Table 2).

#### Protein precipitation

Briefly, 1.2 mL of sample was added to a 2-mL reaction tube containing 1 g of silica beads (0.5 mm, Carl Roth, N030.1) and lysed using a bead mill (30 min, 30 Hz) with a subsequent centrifugation step (8000×*g*, 10 min, room temperature). To a new reaction tube, 1 mL of supernatant was transferred, and 1 mL 20% trichloroacetic acid (w/v, Sigma-Aldrich, 99%) was added before the tube was stored for 1 h at 4 °C. Samples were centrifuged (16,400×*g*, 10 min, 4 °C, same for following steps) and precipitated twice (alternating) with ice-cold acetone (80% v/v, VWR, 99.8%) and ethanol (70% v/v, VWR, 99.8%) for 20 min with subsequent centrifugation after each step. The dried samples were resuspended afterward in 200 μL urea buffer (8 M urea (Applichem) in 0.1 M Tris–HCl, pH 8.5).

#### Tryptic digestion

For tryptic digestion of the proteins, a slightly modified version of the filter-aided sample preparation protocol (Wisniewski et al. 2009) was used. Briefly, samples were loaded on a 1.5-mL reaction tube containing a 10-kDa filter unit (Pall Nanosep, VWR, 516-8492) and treated with 40 mM dithiothreitol (20 min, 300 rpm, 56 °C) and 55 mM iodacetamide (20 min, 300 rpm, room temperature) with subsequent centrifugation steps (10.000×*g*, 10 min, room temperature, same for the following steps). The filter was then washed once with 100 μL of 8 M urea buffer and thrice with 5 mM ammonium bicarbonate. For tryptic digestion, 0.2 mL of a trypsin solution (2.5 μg/mL trypsin in ABC buffer; trypsin MS approved, Serva) was added to the filter. Finally, the samples were incubated over night at 37 °C and 750 rpm in a thermomixer. After centrifugation, the filter was washed with 50 μL extraction buffer (ABC buffer + 5% LC-MS grade acetonitrile) and 50 μL LC-MS grade water and the flow-through of all three steps collected and dried in a vacuum centrifuge (Vacuubrand).

#### Liquid chromatography and MS

Dried peptides were resuspended in 20 μL of chromatographic mobile phase A (LC-MS-grade water, 0.1% trifluoroacetic acid, 99.9%). Of each sample, 2 μL was injected and separated by an UltiMate® 3000 nano splitless reversed phase nanoHPLC (Thermo Fisher Scientific) equipped with a reversed-phase trap column (nano trap cartridge, 300 μm i.d. × 5 mm, packed with Acclaim PepMap100 C18, 5 μm, 100 Å, nanoViper) and a reversed-phase separation column (Acclaim PepMap RSLC, C18, 2 μm, 75 μm, 50 cm). The gradient was 5 to 35% mobile phase B (LC-MS grade acetonitrile, 0.1% formic acid, 99%) over 30 min at a flow rate of 0.4 μL/min. The LC was coupled directly to a timsTOF Pro mass spectrometer (Bruker Daltonik GmbH), equipped with a captive spray ionization source operated in positive ion mode with a capillary voltage of 1400 V and 200 °C capillary temperature. The mass spectrometer was operated in MRM mode mimicking a SWATH-MS (Gillet et al. 2012) strategy. The scan range for MS1 was 400–1000 m/z with 24 isolation windows of 26 m/z (1 m/z overlap), resulting in a total cycle time for a complete scan of the mass range of 3.2 s. The collision energy spanned from 27 to 48 eV (slope = 0.042; intercept = 9.45). The scan range for MS2 was 150–2200 m/z.

#### Data analysis with Skyline

Bruker timsTOF Pro raw files were analyzed using the open-source software Skyline (vs. 19.1) (MacLean et al. 2010). Full-scan settings were applied to reflect the SWATH-MS acquisition parameters described above. Other settings were enzyme = trypsin, maximal missed cleavages = 0, structural modifications = carbamidomethyl (cysteine), isotope label type = heavy. Quantification of peak areas was based on 2-fold charged precursor and singly-charged *y*-ions (y-ion 3 to last ion). Peak borders were manually adjusted.

#### Estimation of M1-OP7 abundance and proportion to total M1

The point mutations of OP7 in Seg7 vRNA result in the expression of a mutated M1 protein (M1-OP7). To quantify the abundance of M1-OP7, a peptide containing one of the OP7 mutations (EITFYGAK) was measured. For quantification of the abundance of total M1, a peptide unaffected by OP7 point mutations (TRPILSPLTK) was measured. For the estimation of the proportion of M1-OP7 to total M1, the abundance of M1-OP7 was divided by the respective abundance of total M1. Subsequently, results were normalized to their corresponding maximum value.

### UV irradiation

Infectious STVs in the produced OP7 material were inactivated using UV light. To expose the produced material directly to the UV light while keeping a sterile environment, a laminar hood was used (Thermo Fisher Scientific, Safe 2020). For inactivation, 45 mL produced material was transferred into a tray (250 cm^2^ surface area) and continually shaken with a mixer (Duomax 1030, 543-32205-00 Heidolph). This ensured a thin film layer (approximately 2 mm) and a homogenous inactivation of the material.

### Innocuity assay

For the identification of residual infectious STV in the UV-irradiated material, an innocuity assay was conducted. Adherent MDCK cells were cultivated in T75-flasks to a confluence of about 80%. After washing, cultivation medium was replaced with 45 mL infection medium. A sample volume of 100 μL was added. Cells were incubated for 72 h. Next, 1 mL of the supernatant was transferred to another T75-flask containing 45 mL of infection medium and incubated for 72 h. Finally, the hemagglutination assay was used to test whether a titer could be detected after the second passage. Samples were subjected to the innocuity assay in triplicates and a positive control (active PR8-RKI), and PBS as negative control was used.

### Downstream processing of OP7 material

A general overview of the production and purification workflow for OP7 is shown in supplementary Fig. S1.

#### Sample preparation before capture chromatography

The UV-inactivated virus harvest was clarified by a series of successive microfiltration steps with regenerated cellulose filter discs with pore sizes of 1.0 μm, 0.45 μm, and 0.2 μm (GE Healthcare) employing a reusable bottle top device (no. 528199-325; VWR) and a vacuum pump. The volume of the filtered sample after the final 0.2 μm step was approximately 450 mL. This sample is named “clarified virus harvest” hereafter.

The host cell DNA in the clarified virus harvest was enzymatically digested using an unspecific nuclease (Denarase®, named “Denarase” hereafter, no. 2DN100KU99; Sartorius Stedim Biotech). The DNA digestion was performed by supplementing the clarified virus harvest with magnesium chloride (no. M8266-1KG; Sigma-Aldrich Chemie GmbH) to a final concentration of 2 mM and 50 U/mL of Denarase. The sample was incubated at room temperature for 4.5 h under mixing with a magnetic stirrer at 250 rpm.

#### Chromatographic purification

All chromatography experiments were performed with an ÄKTA Pure 25 (GE Healthcare) liquid chromatography system. The UV absorbance was monitored at 280 nm, and virus particles were monitored with a NICOMPTM 380 (Particle Sizing Systems) submicron particle analyzer at 632.8 nm. All chromatography experiments were performed at room temperature.

Virus particles were purified by membrane-based SXC as reported previously (Marichal-Gallardo et al. 2017). The SXC filter device (hereafter named “column”) consisted of a stack of 1.0 μm regenerated cellulose membranes (no. 10410014; GE Healthcare) (20 layers; 100 cm^2^ total surface) fitted into a commercial 25-mm stainless steel filter housing as described before. The flow rates used were 5–10 mL/min.

SXC-purification was performed in bind-elute mode. Briefly, (a) Equilibration: the column was washed with 10 column volumes of water followed by 10 column volumes of 8% PEG-6000, PBS. (b) Sample injection: The clarified virus harvest after DNA digestion was mixed in-line to a ratio of 1:1 with a stock solution consisting of 16% PEG-6000 (no. 81260-5KG; Sigma-Aldrich Chemie GmbH) and PBS to achieve a final concentration of 8% PEG-6000 when fed to the SXC column. Sample injection was followed by a wash step with 8% PEG-6000, PBS until baseline UV absorbance was achieved. (c) Elution: virus particles were recovered by washing the filter device with around 20 column volumes of PBS.

Additionally, analytical-size exclusion chromatography (SEC) was performed with a packed-bed Superdex 200 Increase 10/300 GL (no. 17517501; GE Healthcare) column. The sample injection volumes ranged 100–500 μL, and the flow rate was 0.75 mL/min.

#### Differential centrifugal sedimentation

Particle size distribution analysis was estimated by differential centrifugal sedimentation (DCS) as reported previously (Pieler et al. 2017). A CPS DC24000 UHR disc centrifuge was used (CPS Instruments Inc.) at 24,000 rpm with a 4–16% (m/v) sucrose gradient in PBS. The gradient was formed by nine 1.6-mL steps with different sucrose concentration each, i.e., 16%, 14.5%, 13%, 11.5%, 10%, 8.5%, 7%, 5.5%, and 4% sucrose (m/v), with a total volume of 14.4 mL. The gradient quality was evaluated by injecting a 239-nm particle standard (0.3–0.5% solid content, polyvinyl chloride, CPS Instruments Inc.) directly after generating the gradient. The gradient was then equilibrated for 10 min, followed by another injection of a 239-nm particle standard for measurement calibration. Finally, 100 μL of sample (1:1) was injected for the size distribution measurements of samples. Additional density parameters for solutions and particles introduced into the centrifuge software were 1.072 g/cm^3^ for the gradient buffer, 1.385 g/cm^3^ for the calibration particles, and 1.180 g/cm^3^ for IAV. The particle size distribution is displayed as normalized weight average in percentage against apparent hydrodynamic diameter in nm.

#### Formulation

The SXC eluate was dialyzed against PBS overnight at 4 °C with a 100-kDa molecular mass cut-off membrane as described before (Marichal-Gallardo et al. 2017). The collected sample was spiked with sucrose to a final concentration of 1%, followed by a microfiltration step with a 0.2-μm cellulose acetate syringe filter (no. 16534----------K; Sartorius Stedim Biotech) to ensure sterility. This purified OP7 material was stored at – 80 °C until evaluation in *in vitro* assays.

### Mouse infections

For evaluation of the antiviral potential of OP7 *in vivo*, animal experiments in the mouse IAV infection model were performed.

#### Challenge virus generation

Mouse-adapted IAV PR8 virus was generated by plasmid rescue as described before (Hoffmann et al. 2000; Lambertz et al. 2018). For mouse infections, virus was propagated in the chorioallantoic cavity of 10-day-old specific pathogen-free (SPF) embryonated chicken eggs (Charles River Laboratories) for 48 h at 37 °C, aliquoted and stored at – 80 °C. The titer of the stock viruses was determined by focus forming unit assay (FFU/mL) as described (Lambertz et al. 2018) (see Table 2). The identity of the virus was confirmed by next-generation sequencing as described (Lambertz et al. 2018).

#### Infection of mice

DBA/2JRj (D2-*Mx1*^*−/−*^*)* mice carrying a non-functional *Mx1* gene were obtained from Janvier. In our laboratory at the Helmholtz Centre for Infection Research, we generated D2(B6).A2G-*Mx1*^*r/r*^ (D2-*Mx1*^*r/r*^) mice carrying a functional *Mx1* gene. Here, we backcrossed DBA/2JRj mice for 10 generations onto congenic B6.A2G-*Mx1*^*r/r*^ mice provided by Peter Stäheli, University of Freiburg, Germany, as described previously (Shin et al. 2015). All mice were maintained under specific pathogen-free conditions at the Central Animal Facilities of the HZI, Braunschweig. For infection, D2-*Mx1*^*r/r*^ mice (female, 8–12 weeks old) were anesthetized by intra-peritoneal injection of ketamine-xylazine solution (5 mg/mL ketamine, WDT; 1 mg/ml xylazine, CP Pharma; in sterile 0.9% NaCl, WDT) with a dose adjusted to the individual body weight (200 μL/20 g body weight). For evaluation of OP7 toxicity or OP7 antiviral potency, mice were treated with 20 μL solution PBS containing the indicated dose of the tested OP7 material and the indicated dose of STV by intranasal application. Subsequently, body weight and survival were monitored for 14 days. In addition to mice that were found dead, animals with a body weight loss of more than 30% of the starting body weight were euthanized and recorded as dead.

## Results

### OP7 production yield in batch mode is dependent on MOI

**Fig. 1:**
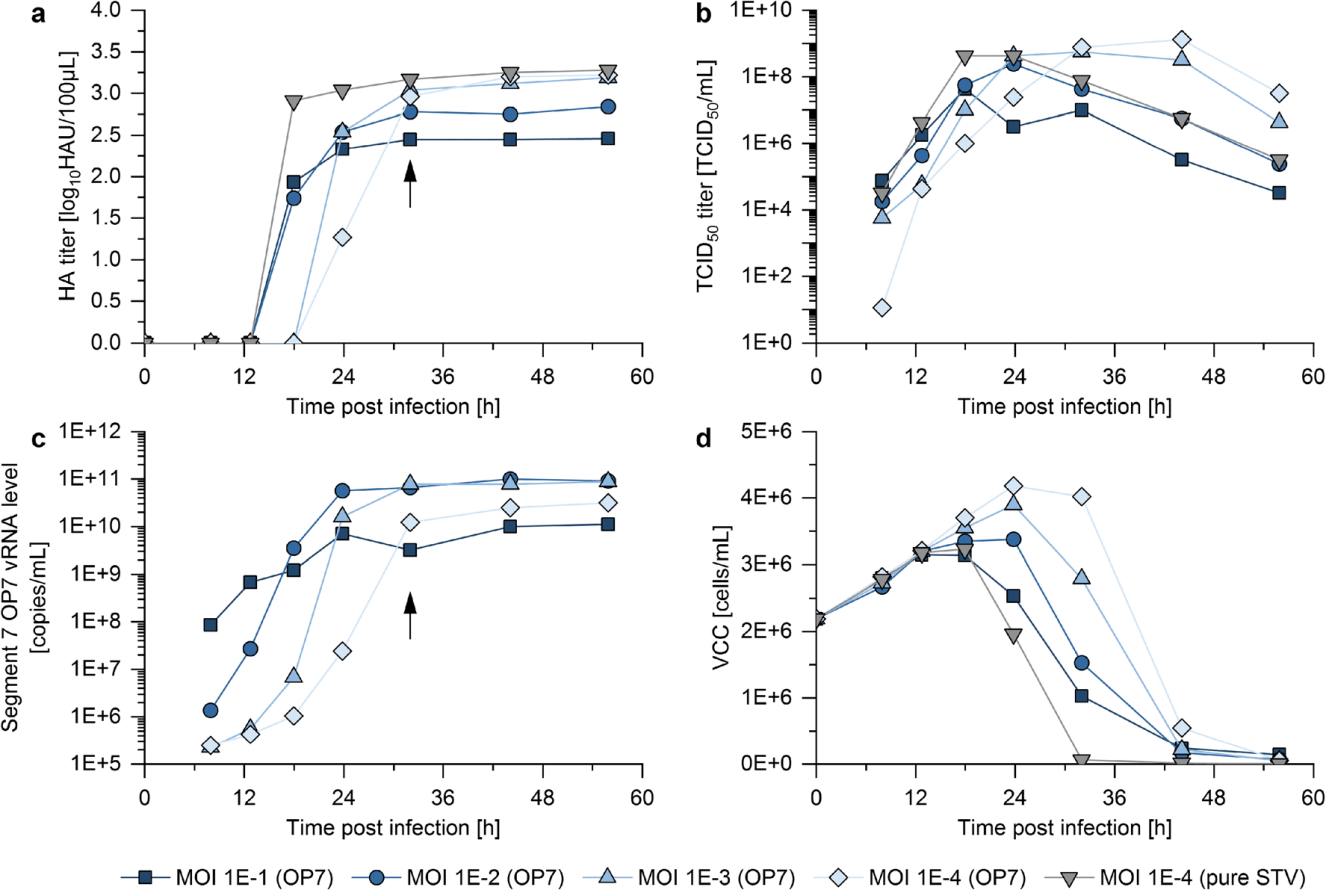
MOI screening for batch-mode production of OP7. Suspension MDCK cells were cultivated in Xeno™ medium in shake flask with 50-mL working volume. Cells were then infected in exponential growth phase at a VCC of 2E+6 cells/mL with OP7 seed virus at different MOIs or pure STV at a MOI of 1E−4. The **a **HA titer, **b **TCID_50_ titer, **c **vRNA level of Seg7 OP7, and **d **VCC are shown. For the pure STV infection, no Seg7 OP7 could be detected. The black arrows indicate the chosen harvest time point (32 hpi) for the following experiments. Illustration includes results of one experiment. Note that additional cultivations harvested at the same time point showed reproducible results (supplementary table S3)

A seed virus containing OP7 and STV was used for cell culture–based production, since propagation of the apparently defective OP7 relies on STV complementation. On the one hand, we expected more STV and DIP co-infections at higher MOIs, likely leading to an enhanced propagation of OP7 over STV. However, it has to be considered that DIP co-infections also suppress virus replication, which may lead to an overall reduced virus titer (and thus, reduced OP7 yield) at higher MOIs. On the other hand, low MOIs should result in higher total virus titers, since cells are mostly infected by single virus particles. Here, only STV-infected cells (but not OP7-infected cells) will produce progeny virions. Therefore, OP7 propagation and interference with virus replication was expected to cease. To test this supposed dependency of OP7 yield on MOI, four MOIs ranging from 1E−1 to 1E−4 were investigated in previously optimized shake flask batch cultures (Bissinger et al. 2019; Genzel et al. 2013; Granicher et al. 2019; Lohr et al. 2010). Suspension MDCK cells in Xeno™ medium were infected with OP7 seed virus. The applied MOI was calculated based on TCID_50_ measurements of the seed virus determining the amount of infectious STV particles. VCC, HA titers (indicating total virus particle concentration), TCID_50_ titers, and vRNA levels of the mutated Seg7 OP7 of released virus particles were quantified over time post infection (Fig. 1).

Indeed, cell cultures infected at higher MOIs resulted in lower HA titers compared with those infected at lower MOIs (Fig. 1a), indicating an interfering effect of OP7 on virus propagation. In line with this observation, maximum TCID_50_ titers were lower with higher MOIs (Fig. 1b). The vRNA levels of Seg7 OP7 showed a higher initial level for higher MOIs (Fig. 1c); yet, the highest maximum vRNA levels were reached for infections at a MOI 1E−2 and 1E−3. As a control, one cultivation was infected with a pure STV seed (devoid of OP7) at a MOI of 1E−4. As expected, the STV infection showed the fastest decrease in VCC (Fig. 1d), while resulting in the fasted increase in HA and TCID_50_ titers.

For all infections, the TCID_50_ titer decreased at late process times, in line with previous findings (Genzel et al. 2010). For instance, the infectious virus titer of the pure STV infection showed a decrease of approximately three orders of magnitude from 24 to 56 h post infection (hpi). Since it needs to be assumed that OP7 particles may lose biological activity similarly, it was considered to harvest as early as possible. A harvest time point of 32 hpi was chosen, where the HA titer and Seg7 OP7 vRNA level roughly reached their respective maximum value (Fig. 1a and c, black arrow). At the same time, TCID_50_ titers were close to their maximum. TCID_50_ titer, HA titer, and Seg7 OP7 vRNA level for the different MOIs at 32 hpi are shown in supplementary table S3 (production replicate 1). Please note that the results shown here include a single experiment. However, independent production replicates (required for additional experiments described in the following sections) showed reproducible results (supplementary table S3). Additionally, OP7 material was produced in a stirred tank bioreactor at a MOI 1E-2. Despite the different production systems, very comparable replication dynamics and maximum virus titers were observed (supplementary Fig. S2).

In summary, it appears that the MOI strongly affects the yield of OP7 (i.e., Seg7 OP7 vRNA). High MOIs resulted in lower virus titers, indicating suppression and interference of virus production by OP7. On the other side, low MOIs resulted in higher total virus particle concentrations. Therefore, intermediate production MOIs (i.e., MOI 1E−2 or 1E−3) seem to allow for high-yield OP7 production due to a good balance between Seg7 OP7 vRNA replication and an acceptable suppression of overall virus production.

### Interfering efficacy of the OP7 material depends on the production MOI

To determine the production MOI yielding the highest biological efficacy, OP7 samples were compared in an *in vitro* interference assay. Here, the suppression of STV replication by OP7 co-infection was assessed. More specifically, STV infected cells were co-infected with OP7 material, and the reduction of released infectious virus particles was compared with those of STV only infected cells. STV was added at MOI 10 and harvested OP7 material with a fixed volume of 125 μL in order to identify a production condition showing the highest interfering efficacy per product volume. The TCID_50_ titer, HA titer, and OP7 Seg7 vRNA level for the OP7 preparations produced with different MOI are shown in supplementary table S3 (production replicate 2).

**Fig. 2:**
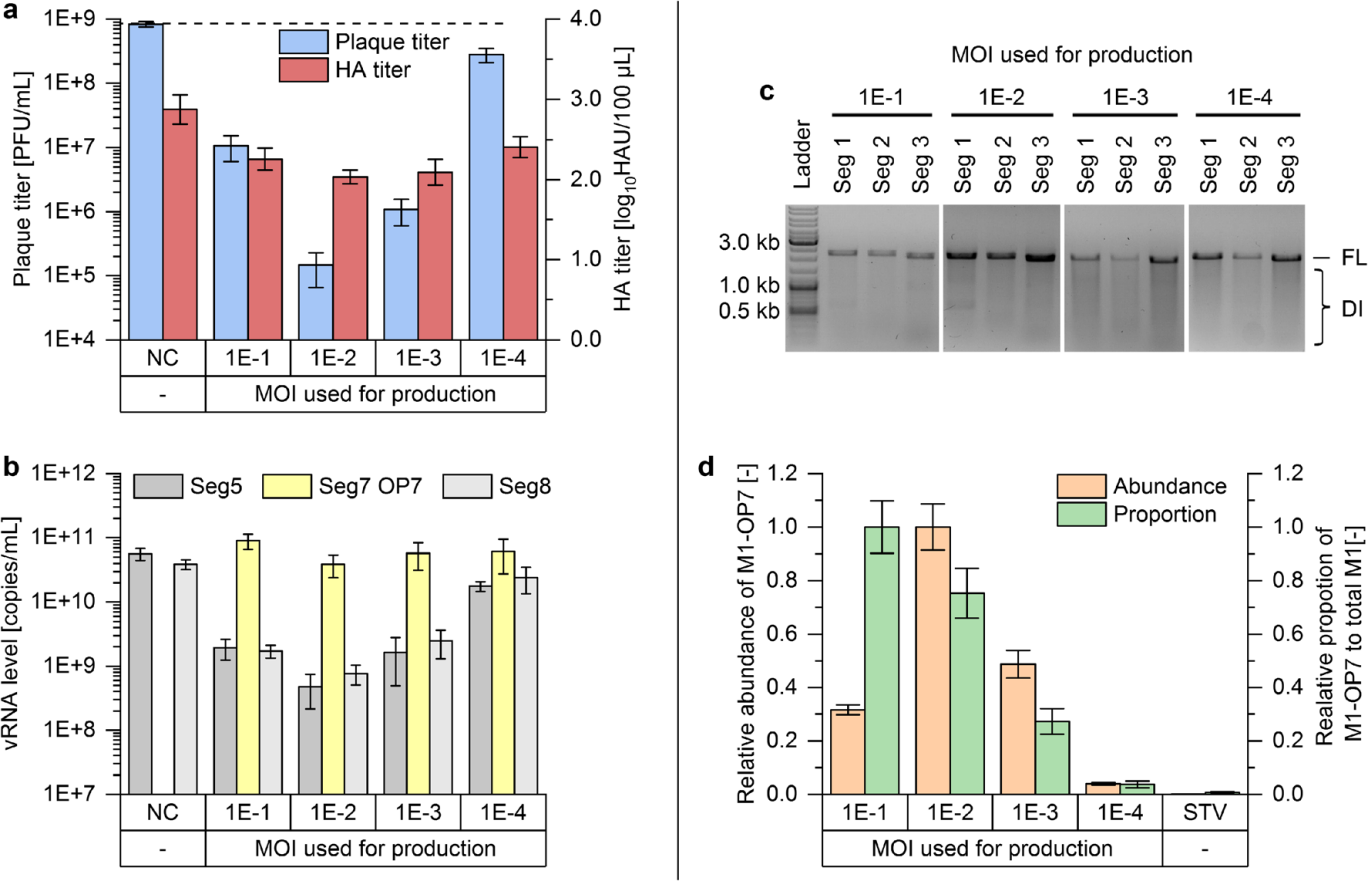
Evaluation of OP7 material produced at different MOIs. **a**, **b** Interference assay. Adherent MDCK cells were infected at a MOI of 10 with STV and co-infected with 125 μL of OP7 material, produced at a MOI ranging from 1E−1 to 1E−4 or medium as NC. At 16 hpi, **a **virus titers and **b **vRNA levels of Seg5, Seg7 OP7, and Seg8 in the progeny virions were determined. **c**, **d** OP7 materials, produced at different MOIs. **c **Results of segment-specific reverse transcriptase-PCR. Signals corresponding to full-length (FL) and DI vRNAs of Seg1, Seg2, and Seg3 are indicated. **d **Results of MS. The relative abundance of M1-OP7 and the relative proportion of M1-OP7 to total M1 protein are shown. Results were normalized to the respective maximum value. As a control, a plaque-purified STV, devoid of OP7, was measured. The interference assay was performed in independent experiments (*n* = 3) using one OP7 production sample. MS measurements were performed as technical replicates (*n* = 3) of one production sample. Error bars indicate standard deviation

Figure 2a shows the results of the interference assay. OP7 material produced at a MOI of 1E−2 showed the strongest interference, reducing the infectious virus titer (determined by plaque assay) by almost four orders of magnitude. A very comparable titer reduction was observed for material produced at MOI 1E−2 in a stirred tank bioreactor (supplementary Fig. S2). Material produced at a MOI of 1E−1 or 1E−3 showed a less pronounced titer reduction, and OP7 produced at MOI 1E−4 decreased the plaque titer just marginally. A similar trend could be observed for the reduction in total virus particle release, as indicated by the HA titer. The observed differences are not as pronounced, as the release of non-infectious OP7 particles also contributes to total virus particle concentrations.

The interference of OP7 was further assessed in vRNA measurements of the released virus particles using real-time RT-qPCR (Fig. 2b). First, material produced at any MOI induced an OP7 phenotype in the interference assay, indicated by an over-proportional level of Seg7 OP7 vRNA, as described previously (Kupke et al. 2019). While the maximum Seg7 OP7 vRNA levels were comparable, Seg5 and Seg8 vRNA levels showed a stronger reduction at higher interference.

To ensure that observed interfering effects were not caused by conventional DIPs, a segment-specific RT-PCR was performed. The method was previously used to detect deleted (and short) DI vRNAs of IAV of segment 1–8 (Seg1–8) in cell culture (Frensing et al. 2013; Tapia et al. 2019). Figure 2c shows the results for Seg1, Seg2, and Seg3, which are most prone for the formation of conventional DI vRNAs. DI vRNAs were observed neither on these segments nor on the remaining segments (supplementary Fig. S3) for material produced at any MOI.

The point mutations on Seg7 OP7 vRNA also affect the coding region, resulting in a mutated M1-OP7 protein, which is presumably incorporated into OP7 particles. To investigate this, MS was used to quantify M1-OP7 (Fig. 2d). Here, M1-OP7 was quantified by measuring the abundance of a fragment peptide that carried a mutation in the respective amino acid sequence. The abundance of total M1 protein was quantified by measurements of a fragment peptide that was identical for OP7 and wild-type virus. In line with the results of the interference assay, the relative abundance of M1-OP7 was highest for OP7 produced at a MOI of 1E−2 and lowest for a production MOI of 1E−4. Next, the proportion of the M1-OP7 to total M1 protein was estimated. We observed that higher production MOIs led to higher proportions of M1-OP7, with OP7 produced at MOI 1E−1 showing the highest proportion. However, the total amount of produced M1-OP7 was higher for material produced at MOI 1E−2, which may be explained by the decreased total virus titer for the production MOI of 1E−1 (Fig. 1b). As a control, a plaque-purified STV sample (devoid of OP7) was used, which was tested negative for the presence of M1-OP7. In contrast, the peptide for total M1 protein could be measured for all samples (supplementary Fig. S4), confirming the validity of the measurement.

In summary, the interference assay showed the highest interfering efficacy for material produced at a MOI of 1E−2, in line with MS measurements indicating the highest amount of M1-OP7.

### UV irradiation completely inactivates STV while preserving interfering efficacy of OP7

In order to inactivate STV in the produced OP7 material, which may cause harm in a potential application, UV irradiation was used. Moreover, we targeted at short inactivation times to avoid a potential loss of interfering efficacy of OP7.

**Fig. 3:**
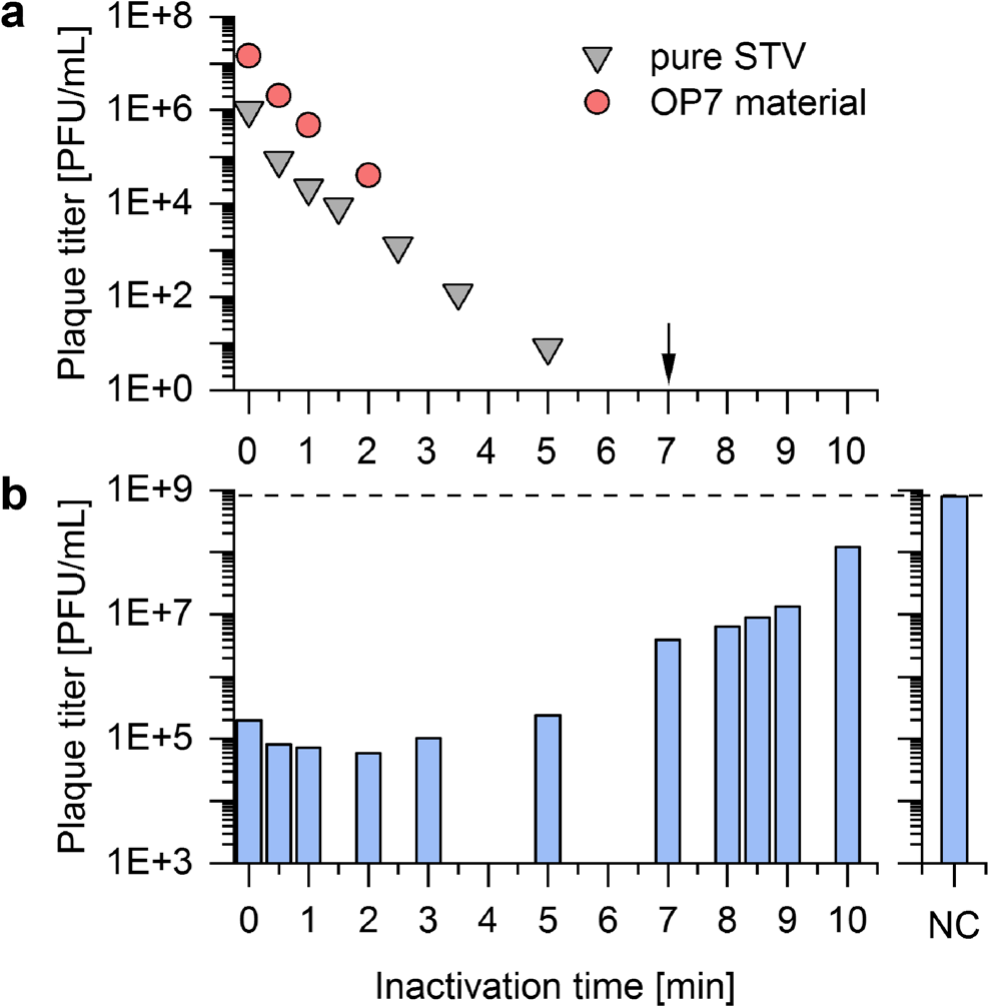
UV irradiation of produced OP7 material. Produced OP7 material (MOI 1E−2, 32 hpi) was irradiated with UV light. **a** Infectious virus titer in the sample and **b** infectious virus titer in the interference assay (described in Fig. 2) were determined for different UV inactivation times, where 0 min corresponds to UV untreated samples. **a **As a control, the decrease in infectious virus titer of a pure STV, devoid of OP7, was tested. The black arrow indicates the first time point where no infectious virus could be detected for the UV-treated OP7 material, as shown by an innocuity assay. **b **For the interference assay, medium was tested as a NC. Panels include results of one experiment

Figure 3a shows the inactivation kinetics of a pure STV sample and of OP7 produced at a MOI of 1E−2. For the STV sample, infectious virus titers steadily decreased with irradiation time until 5 min. At 6 min, the detection limit of the plaque assay was reached. However, for the OP7 material, no infectious virus titer could be detected already after 2 min UV irradiation. Here, it is likely that the quantification of STV in the plaque assay (detecting only infectious viruses) was impeded by the suppression of virus replication by OP7 particles.

Instead, to demonstrate the absence of STV for UV-treated OP7 material, an *in vitro* innocuity assay was performed. Here, a small sample volume was used for infection of a large number of cells, followed by another infection passage after 3 days. This complies with a low MOI scenario in which almost exclusively single-hit infections occur. Accordingly, these infection conditions allow for the propagation of residual STVs, which might have not been detectable by the plaque assay (Fig. 3a). Afterward, virus accumulation was determined by hemagglutination assay. Indeed, OP7 material irradiated up to 6 min was tested positive in the presence of infectious STV. The first time point tested negative was 7 min of UV irradiation (black arrow: Fig. 3a). Nevertheless, for safety reasons, an inactivation time of 8 min was chosen for processing of OP7 material for subsequent animal experiments.

Next, the interfering efficacy was determined for OP7 material inactivated for different UV irradiation times (Fig. 3b). With longer inactivation times, the interfering efficacy decreased, which can be explained by inactivation of OP7 particles. Interestingly, up until 1 min inactivation time, the interfering efficacy slightly increased, which may be explained by a faster inactivation of STV compared with OP7.

Taken together, UV irradiation resulted in a complete inactivation of STV in the produced OP7 material after 7 min. Still, to a large degree, the interfering efficacy of OP7 was preserved.

### Downstream purification and concentration greatly improve interfering efficacy of UV-irradiated OP7 material

**Fig. 4:**
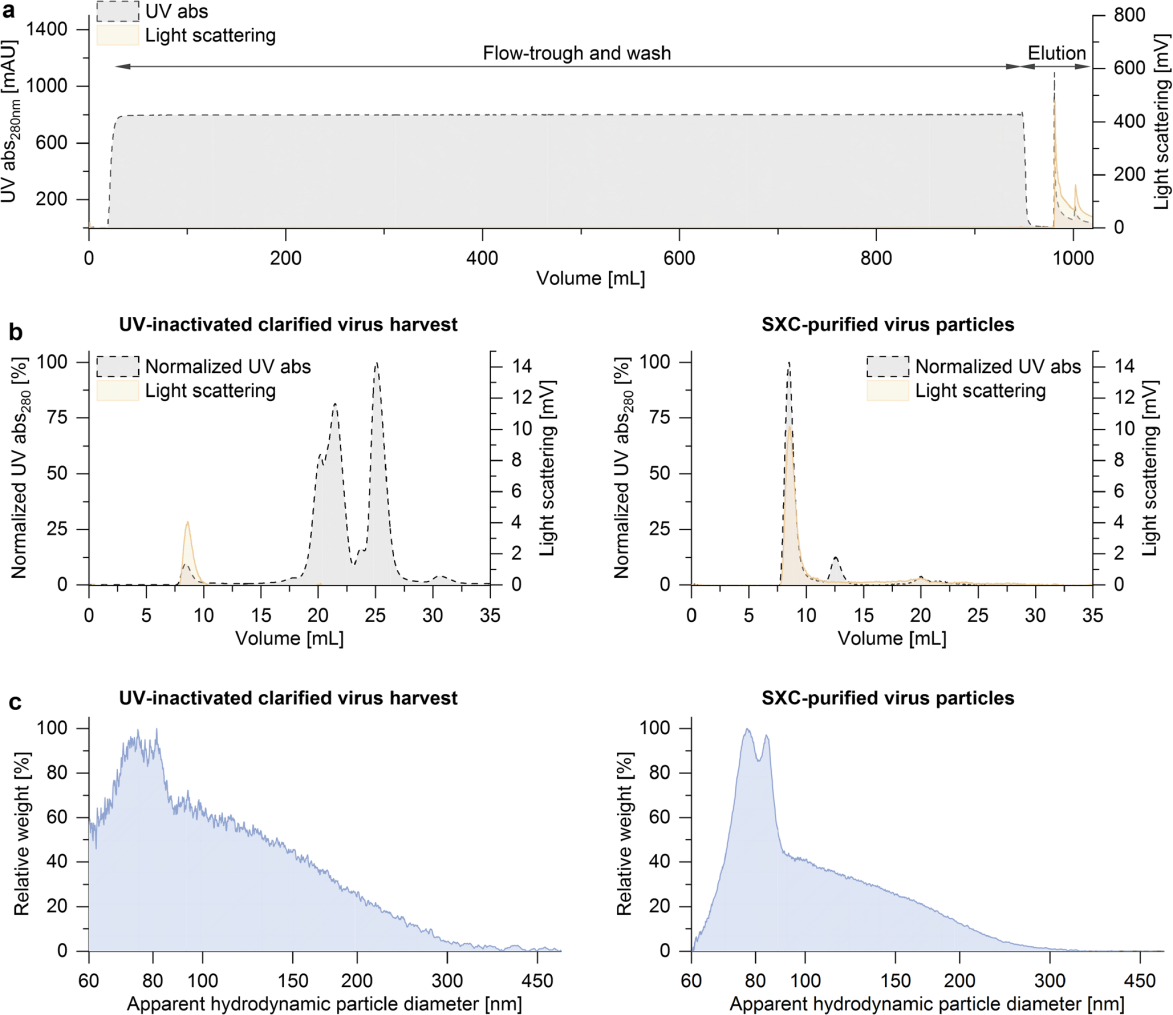
Purification of virus particles by SXC. UV-inactivated OP7 material was purified using membrane-based SXC. (**a**) The light-scattering signal indicates the presence of the IAV particles in the elution step. (**b**) Analytical size-exclusion chromatography fingerprints of IAV samples before and after membrane-based SXC. The virus purity based on the UV signal was 2.7% for the clarified virus harvest (left panel) and 89.1% for the SXC-purified material (right panel). (**c**) Particle size distributions determined by differential centrifugal sedimentation. The signal noise of the unpurified sample in the left panel is due to a lower particle concentration compared with the SXC-purified material on the right panel. The purification was performed in a single experiment

In order to increase the volumetric interfering efficacy, the UV-irradiated OP7 material was purified and concentrated by membrane-based SXC. Therefore, the UV-inactivated and DNA-digested clarified virus harvest was mixed in-line 1:2 with 16% PEG-6000 and fed to a chromatography filter unit. Based on the SXC chromatogram shown in Fig. 4a, no visible losses of virus particles were observed in the flow-through during sample loading and column wash, evidenced by a nil light scattering signal. The absence of IAV particles in the flow-through fraction was confirmed by the hemagglutination assay. The purified virus particles were eluted in PBS, and the product yield estimated with the hemagglutination assay was 45.2% relative to the load (further discussion on product yield in “Purification of processed OP7 material”).

Figure 4b shows analytical SEC fingerprints of the clarified virus harvest (left panel) and the SXC-purified virus particles (right panel). The virus particles are traced by light scattering at a retention volume of 7.5–10 mL. The virus purity based on the total protein detected by the UV signal at 280 nm from the SEC fingerprints was 2.7% for the clarified virus harvest and 89.1% for the SXC-purified sample (area under the curve of the virus peak divided by the total area of the chromatogram).

Figure 4c shows the particle size distributions of the unpurified and purified samples determined by DCS analysis (left and right panel, respectively). The signal noise of the unpurified sample is due to a lower particle concentration compared with the purified material. Two main peaks are observed at around 80–90 nm with the additional presence of less concentrated larger particles up to 250 nm.

**Fig. 5:**
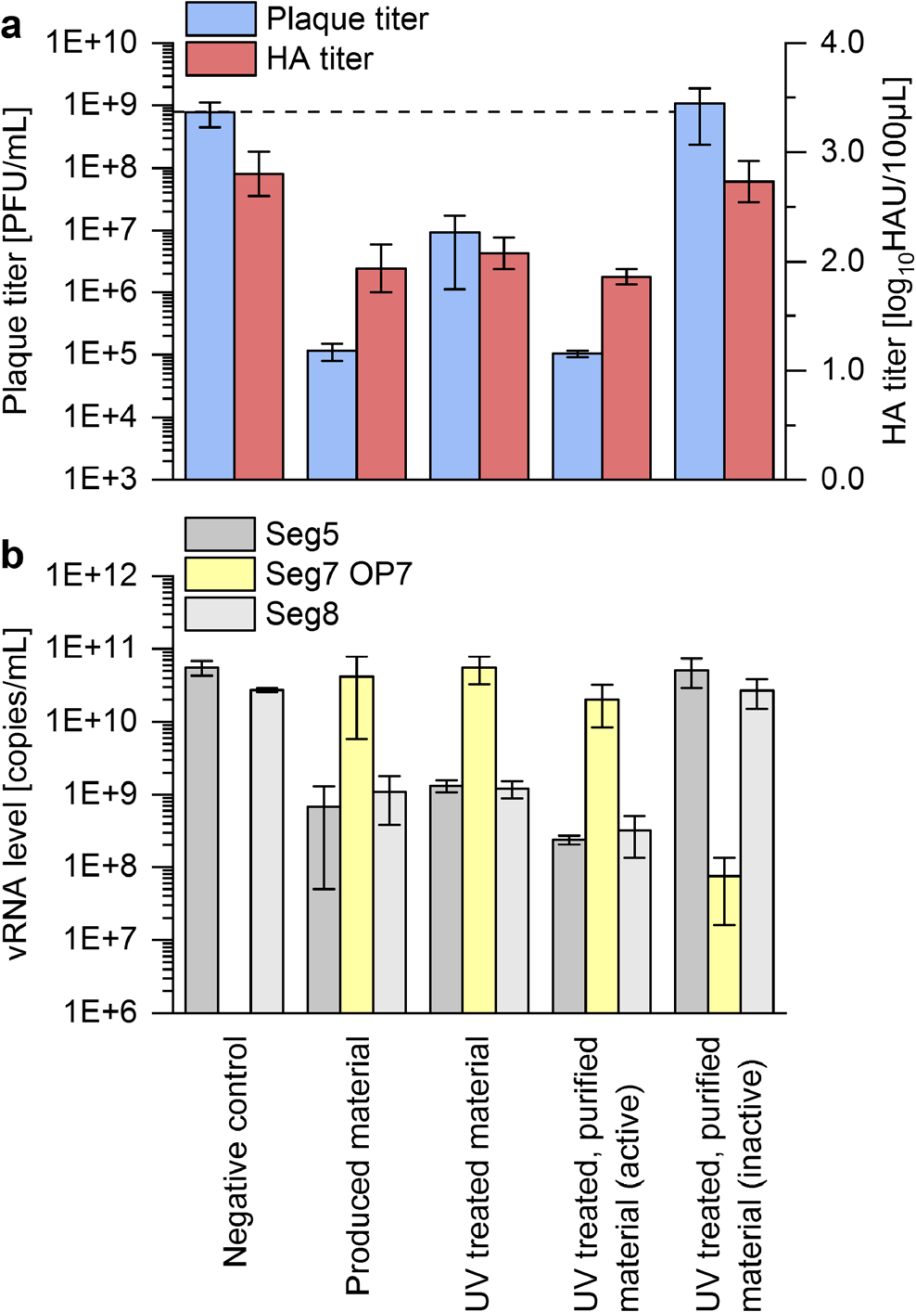
Interfering efficacy of processed OP7 material. An interference assay was conducted as described in Fig. 2. The produced OP7 material; produced material irradiated with UV (for 8 min); UV-treated, SXC-purified material (active); and SXC-purified material, inactivated by UV light for 24 min (inactive), and medium as negative control was tested. **a **Virus titers and **b **vRNA levels of Seg5, Seg7 OP7, and Seg8 in the progeny virions are shown. The interference assay was performed in independent experiments (*n* = 3), each using one preparation. Error bars indicate standard deviation

Next, the concentrated and SXC-purified (1.12E+11 Seg7 OP7 vRNA copies/mL) material was analyzed in the interference assay (Fig. 5). Although the produced material was treated by UV irradiation, SXC purification resulted in a greatly increased interfering efficacy (Fig. 5a). The UV-irradiated, SXC-purified OP7 reduced the release of infectious virus particles by approximately four orders of magnitude, similar to the non-concentrated OP7 material without UV irradiation. In line with this, the vRNA levels of Seg5 and Seg8 in the released virus particles show the strongest reduction for infection with the purified OP7 material (Fig. 5b). The increased volumetric interfering efficacy after the SXC-purification was most likely caused by the approximately 13-fold increase in the concentration of total virus particles, as indicated by HA titers, which showed an increase from 2.61 to 3.67 log_10_ HAU/100 μL. An inactivated OP7 control was generated by UV irradiation (for 24 min) of the purified material. This material did not show an interfering effect, probably due to complete inactivation of OP7. Indeed, only residual amounts of Seg7 OP7 vRNA could be detected in the released virus particles.

In summary, UV-irradiated OP7 material was purified by SXC, resulting in an approximately 13-fold concentration and a greatly improved interfering efficacy.

### OP7 material protected mice against a lethal dose of IAV upon co-administration

Studies in a mouse infection model were performed to evaluate the antiviral effect of OP7 material *in vivo*. The tested material was cell culture–derived unpurified OP7, UV-inactivated for 8 min to inactivate potentially harmful STV; this material is referred to as “active OP7” (0 TCID_50_/mL, 2.02 E+10 Seg7 OP7 vRNA copies/mL). As negative controls, OP7 material that was UV inactivated for 24 min was used. This material is referred to as “inactive OP7.”

**Fig. 6:**
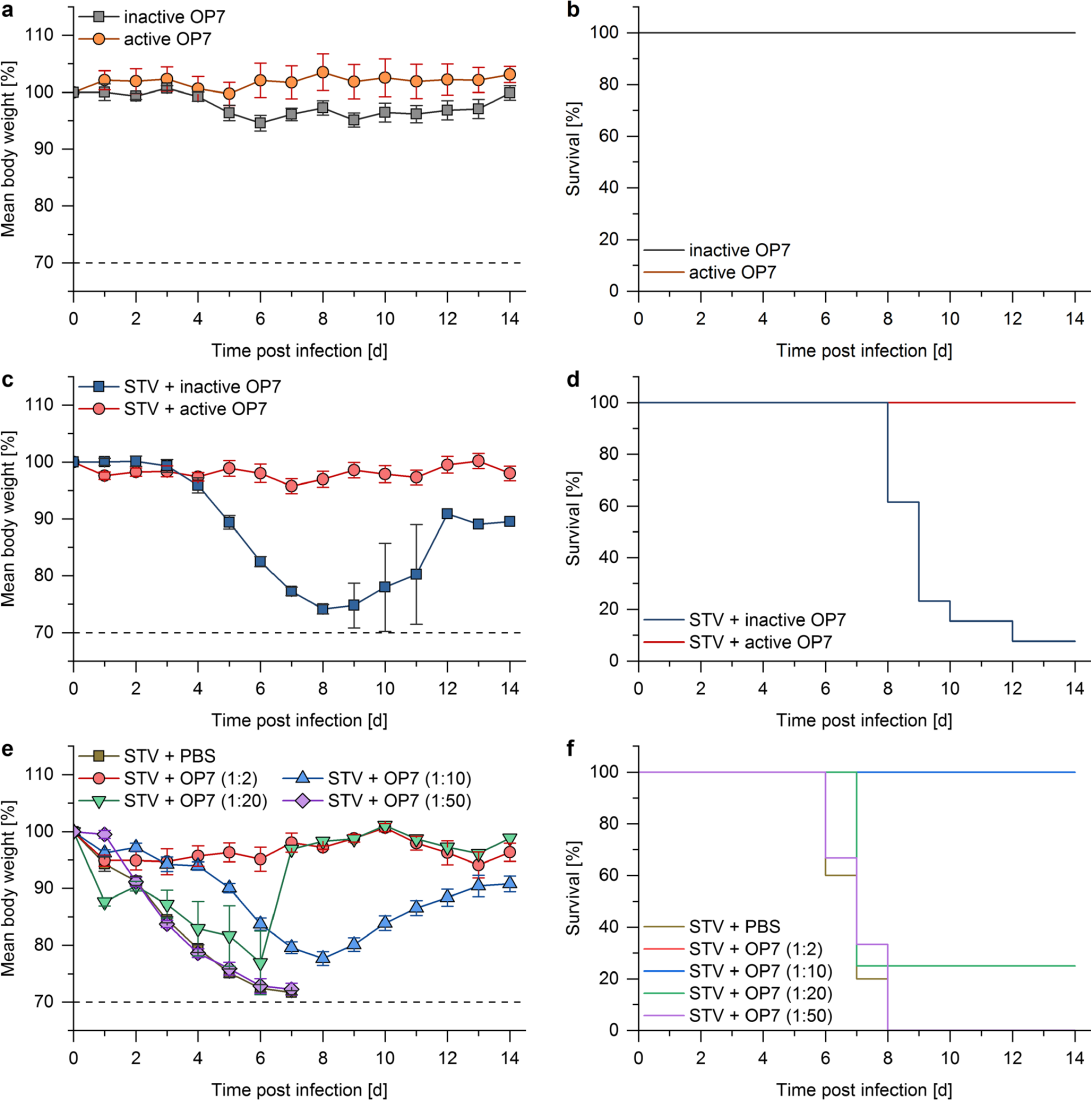
Mouse infection experiments with OP7 material. Female 8–12-week-old D2-*Mx1*^*r/r*^ mice were intra-nasally infected with 20 μL solution containing OP7 and/or STV in PBS. **a**, **b** Toxicity was tested by applying 10 μL active (*n* = 5) or inactive (*n* = 5) OP7 material individually (dose: 2.2E+8 Seg7 OP7 vRNA copies/mice). All mice survived the treatment with active or inactive OP7. **c**, **d** Mice were co-treated with 10 μL active (*n* = 9) or inactive (*n* = 13) OP7 material (dose: 2.2E+8 Seg7 OP7 vRNA copies/mice) and a lethal dose of 1000 FFU STV. **e**, **f** Mice were infected with a lethal dose of 1000 FFU STV and treated with different dilutions of active OP7 (1:2, dose: 2.2E+8 Seg7 OP7 vRNA copies/mice (*n* = 4); 1:10, dose: 4.4E+7 Seg7 OP7 vRNA copies/mice (*n* = 6); 1:20, dose: 2.2E+7 Seg7 OP7 vRNA copies/mice (*n* = 4); 1:50, dose: 8.8E+6 Seg7 OP7 vRNA copies/mice (*n* = 6)) or PBS (*n* = 10). All mice treated with active OP7 diluted 1:2 or 1:10 survived the infection. *Y*-axes show percentages (in relation to day 0) of mean body weight losses (**a**, **c**, **e**) and survival rates (**b**, **d**, **e**) for each group. Error bars indicate one standard error of the mean (SEM) for body weight changes

First, toxicity of the material was examined (Fig. 6a and b). For this, OP7 material (active or inactive; 2.2E+8 Seg7 OP7 vRNA copies/mice) was intranasally applied to mice. Neither active OP7 nor inactive OP7 caused body weight loss, and all mice survived the treatment. These results demonstrate that OP7 alone did not cause any obvious toxic effects.

Next, mice were infected with a lethal dose of 1000 FFU STV (Fig. 6c and d) and co-treated with active OP7 or inactive OP7 (2.2E+8 Seg7 OP7 vRNA copies/mice). Mice treated with inactive OP7 started to lose weight 4 days post infection, and all but one mice died. In strong contrast, mice treated with active OP7 showed no body weight loss, and all the animals survived the infection.

Finally, different concentrations of active OP7 ranging from 2.2E+8 Seg7 OP7 vRNA copies/mice (1:2) to 8.8E+6 Seg7 OP7 vRNA copies/mice (1:50) were tested for their antiviral effect (Fig 6f and e). Treatment with 1:2 diluted active OP7 resulted in a 100% survival rate and no significant body weight loss. Mice treated with 1:10 diluted active OP7 lost weight but all recovered and survived the infection. Twenty-five percent of mice treated with 1:20 diluted active OP7 still survived the infection. However, when active OP7 was diluted 1:50, no difference to PBS treated IAV-infected mice could be detected, and all the mice died 8 days post infection.

Taken together, treatment of mice with active or inactive OP7 material did not show any obvious toxic effects. Moreover, co-treatment with active OP7 protected mice against a lethal dose of STV. Here, the minimum required dose to achieve a 100% survival rate was 4.4E+7 Seg7 OP7 vRNA copies/mice (1:10 dilution). Mice treated with an OP7 dose as low as 2.2E+7 Seg7 OP7 vRNA copies/mice (1:20 dilution) were still partially protected. These results clearly demonstrate the antiviral potential of OP7.

## Discussion

In this study, a cell culture–based production process for OP7 was established. The produced material was processed via UV irradiation and purified with SXC. This purified material showed strong interfering efficacy in a cell-based *in vitro* interference assay. Animal experiments with mice showed a 100% rescue of mice infected with a lethal dose of IAV upon OP7 co-treatment, demonstrating its potential for antiviral therapy.

### Identification of the production conditions yielding a high interfering efficacy

Cell culture–based production of IAV in shake flasks was previously established in our group for vaccine manufacturing (Bissinger et al. 2019; Genzel et al. 2013; Granicher et al. 2019; Lohr et al. 2010). Therefore, many parameters including media composition, shaking orbit, and stirrer speed were already optimized. Here, we investigated the impact of the applied MOI, the harvest time, and the VCC at time of infection on OP7 yield. The interfering efficacy of material harvested at different time points or produced with different VCC at time of infection did not show any significant differences (data not shown). However, as previous works suggested, the MOI had a large impact on yield (Dimmock et al. 2008; Frensing 2015; Tapia et al. 2019; Wasik et al. 2018). The highest interfering efficacy was observed for material produced at a MOI of 1E−2. This can be explained as follows. On the one hand, the yield of Seg7 OP7 vRNA was very similar for a production MOI of 1E−2 and 1E−3; however, the production MOI of 1E−3 resulted in a higher infectious virus titer. Here, the higher STV concentration may have resulted in a reduced interfering efficacy in this sample, probably by a stronger competition of STV against OP7. As different vRNA segments compete for resources in translation, increased amounts of STV vRNA may have also had negative impact on the amounts of produced M1-OP7. More specifically, MS measurements indicated reduced amounts of M1-OP7 for virions produced at a MOI of 1E−3 compared with those produced at a MOI of 1E-2. Here, it was speculated that M1-OP7 may also contribute to the interference of OP7 (Kupke et al. 2019), which could explain the lower interfering efficacy for material produced at a MOI of 1E-3.

### Comparison of cell culture–based production processes for DIPs

The cell culture–based batch production process suggested here has advantages over a previously reported egg-based process for DIPs (Dimmock et al. 2008). In principle, the cell culture–based process allows for an improved scalability and flexibility. Furthermore, it allows for better defined process conditions and the possibility to monitor the production. Therefore, the production process can be characterized in depth, and reproducible product quality can be ensured (Frensing et al. 2014; Swick et al. 2014; Tapia et al. 2019; Wasik et al. 2018).

Previously, a process for continuous production of DIPs was reported (Frensing et al. 2013; Tapia et al. 2019). However, the here proposed batch process may hold several advantages. For example, the reproducibility and product quality are higher for a batch process. For instance, in continuous cultivation, the *de novo* generation and accumulation of conventional DI RNAs was observed (Frensing et al. 2013; Tapia et al. 2019). This resulted in a mixture of a variety of DIPs and thus a less-defined product. In contrast, no accumulation of conventional DI RNAs was observed in the batch cultivations for any genome segment, probably due to a shorter cultivation time and a lower MOI. Moreover, an oscillating titer and fluctuating DIP to STV ratios were observed in continuous cultivation (Frensing et al. 2013; Tapia et al. 2019), which might complicate the selection of an optimal harvest time point. In contrast, the batch process shown here resulted in very reproducible titers.

The results obtained in this proof-of-concept study should facilitate scale-up and further process improvements. The high comparability of a production in shake flask and a stirred tank bioreactor indicate that production in larger scales is equally feasible. Furthermore, process intensification strategies could be applied similar to those that have already been widely explored for production of monoclonal antibodies in CHO cells (Pollock et al. 2013) or the production of flaviviruses (Nikolay et al. 2018). In the latter, a continuous feeding and harvesting scheme (perfusion) was implemented that used a cell retention device to achieve higher cell concentrations and, subsequently, higher virus titers. For IAV and DIP production, further improvement seems also possible. More specifically, a strategy for continuous harvesting of produced virus particles may be employed. This might result in higher yields of biologically active virions by avoiding the time-dependent degradation and loss of biological activity, which is typically observed in a cultivation (Genzel et al. 2010). However, continuous virus harvest with membrane-based cell retention remains problematic due to potential blocking of the membrane (Genzel et al. 2014). Alternatively, cell retention devices like an acoustic settler might be used for continuous virus harvesting (Coronel et al. 2020; Granicher et al. 2020).

### UV irradiation for inactivation of STV in OP7 manufacturing

For the inactivation of STV in the produced material, a previously described UV-irradiation principle was applied (Dimmock et al. 2008). Here, a faster inactivation of STVs compared with DIPs was expected (Dimmock et al. 2008). In theory, an STV already loses its infectivity when only one segment is inactivated, while OP7 only loses its interfering efficacy when specifically Seg7 OP7 is inactivated. Moreover, Seg7 is rather small (~ 1 kb) compared with, e.g., Seg1, Seg2, and Seg3, with more than 2 kb, decreasing the likelihood of its inactivation. Nevertheless, the UV irradiation inactivated parts of the produced OP7 and resulted in a reduced interfering efficacy. One approach to avoid the need of UV irradiation may be the production of pure OP7 particles using a cell line that complements the defect in replication of OP7. Such a system was already successfully applied for the generation of conventional DIPs carrying a deletion on Seg1 vRNA using a complementing cell line expressing the corresponding missing protein polymerase basic 2 (Bdeir et al. 2019; Yamagata et al. 2019). A similar strategy might be used for the production of OP7, for example, by using a cell line expressing the wild-type M1 and/or M2 protein. Note that previous results suggest that the mutated M1-OP7 may explain or contribute to the defect in the replication of OP7 (Kupke et al. 2019).

### Purification of processed OP7 material

The SXC purification resulted in a product yield of 45.2%, which was lower than the typically observed yield of > 95% for the PR8 and other influenza virus strains purified using the same conditions (Marichal-Gallardo et al. 2018). In our experience, yields lower than 80% when purifying influenza virus particles with SXC are mostly due to fouling of the stationary phase caused by the presence of submicron size particles, as observed in the DCS particle size distribution of the unpurified material. This is further supported by the absence of virus particles in the flow-through. The presence of submicron particles might be minimized by changes during cell culture or sample processing (e.g., harvest time, UV inactivation), and this reduction might lead to higher purification yields.

The two main peaks detected by DCS analysis (80–95 nm) of the SXC-purified sample are consistent with the reported size range for IAV particles of 80–120 nm (Pieler et al. 2017). The difference between both peaks might be due to aggregates or to previously observed size differences between STV and OP7 (Kupke et al. 2019). The particle population in the purified material might be further characterized in future studies by using analytical SEC integrated to multi-angle light scattering, ion exchange chromatography, or isoelectric focusing.

Membrane-based SXC-purification was able to eliminate most UV-detected impurities as evidenced by the analytical SEC fingerprints. Before SXC, the virus peak had an estimated purity of 2.7%, compared with 89.1% after SXC. Further polishing of the sample to achieve higher purity could be done after SXC by using pseudo-affinity chromatography with sulfated cellulose membrane adsorbers (SCMA) (Fortuna et al. 2018). Both of these membrane-based purification methods rely on disposable devices and can be scaled-up by linearly increasing the membrane surface needed.

The separation of STV and DIPs by SXC was not studied in this work. Although separation in SXC is mainly based on particle size, other variables play a role in the separation efficiency, such as pH, conductivity, and the isoelectric point of the target product. The possibility of resolving STV from DIPs using a modified version of the SXC protocol used here is an interesting prospect for future work.

Based on the results obtained in this work, we believe the following options are interesting for future purification experiments: (1) increasing the product yield of SXC by optimizations in cell culture to reduce the presence of submicron particles and/or testing of stationary phases with pore sizes larger than 1 μm; (2) resolving OP7 from STV exploiting physical or biochemical differences between both that will allow their separation (e.g., size, isoelectric point); (3) polishing of SXC eluates with pseudo-affinity chromatography using SCMA; (4) further characterization of trace impurities (host cell DNA, host cell proteins) and their maximum levels once an appropriate definition of a human dose for an OP7 product has been established.

### Evaluation of OP7 interfering efficacy in *in vitro* and animal models

Although the material tested in animal experiments was unpurified cell culture supernatant containing high amounts of impurities, no toxic or pathogenic effects could be observed upon OP7 administration. Most of these impurities could have been removed by SXC, as discussed above, and would result in a better defined and more pure product. Furthermore, SXC would have further improved the interfering efficacy of the OP7 material, as shown by our *in vitro* interference assay. This demonstrates that the antiviral efficacy of the produced OP7 could be further improved if necessary. On the other hand, treatment with unpurified OP7 was already sufficient to protect IAV-infected mice. Treated mice showed no body weight loss, and all survived the infection. This clearly demonstrates the antiviral potential of OP7.

The D2-*Mx1*^*r/r*^ mice used in the present study represent a specific model that mimics the immune response in humans to IAV infections. In humans as well as in mice, the strong resistance gene *Mx1* represents an interferon-induced restriction factor against IAV infections. Most commonly used laboratory mice strains lack *Mx1*. C57BL/6J mice, which carry a functional *Mx1* gene, are highly resistant against IAV infections and therefore not suitable to test the antiviral effects of OP7 (Shin et al. 2015). Therefore, we generated another *Mx1* mouse model on the background of DBA/2J mice. These D2-*Mx1*^*r/r*^ mice express a functional *Mx1* but are still susceptible to IAV infections (Shin et al. 2015). These mice represent an ideal mouse model where, as in humans, *Mx1* confers some but not absolute protection to IAV infections. As a next step, it would also be desirable to conduct infection experiments in ferrets. Ferrets are susceptible to human virus and to air-borne virus transmission. Therefore, ferrets represent another important model to study human influenza (Barnard 2009; Whitley 2010).

## Supplementary information

ESM 1 (PDF 534 kb)

## Data Availability

The datasets generated for this study are online available: 10.17632/6p4shpchdn.1 Cell lines and virus seeds will be made available upon request.
