# Supplementary material for: OP7, a novel influenza A virus defective interfering particle: production, purification, and animal experiments demonstrating antiviral potential
Source: Appl Microbiol Biotechnol. 2020 Dec 4;105(1):129–46. doi: 10.1007/s00253-020-11029-5 (PMC7778630; doi:10.1007/s00253-020-11029-5)
Supplement: Supplementary file 1 — (PDF 534 kb) [file 253_2020_11029_MOESM1_ESM.pdf]

Journal: "Applied Microbiology and Biotechnology"

# Manuscript Title: "OP7, a novel influenza A virus defective interfering particle: production, purification, and animal experiments demonstrating antiviral potential"

**Marc D. Hein[1], Heike Kollmus[2], Pavel Marichal-Gallardo[3], Sebastian Püttker[1], Dirk Benndorf[1,3], Yvonne Genzel[3], Klaus Schughart[2,4,5], Sascha Y. Kupke[3,*], Udo Reichl[1,3]**

[1]Otto-von-Guericke-University Magdeburg, Chair of Bioprocess Engineering, Germany, 39106 Magdeburg, Universitätsplatz 2

[2]Department of Infection Genetics, Helmholtz Centre for Infection Research, Germany, 38124 Braunschweig, Inhoffenstraße 7

[3]Max Planck Institute for Dynamics of Complex Technical Systems, Bioprocess Engineering, Germany, 39106 Magdeburg, Sandtorstraße 1

[4]University of Veterinary Medicine Hannover, Germany, 30559 Hannover, Bünteweg 9

[5]Department of Microbiology, Immunology and Biochemistry, University of Tennessee Health Science Center, USA, Tennessee 38163, Memphis, 920 Madison Avenue

*** Correspondence:**
Sascha Y. Kupke
E-mail: kupke@mpi-magdeburg.mpg.de
Phone: +49 391 6110-253
Fax: +49 391 6110-203

# Supplementary Material

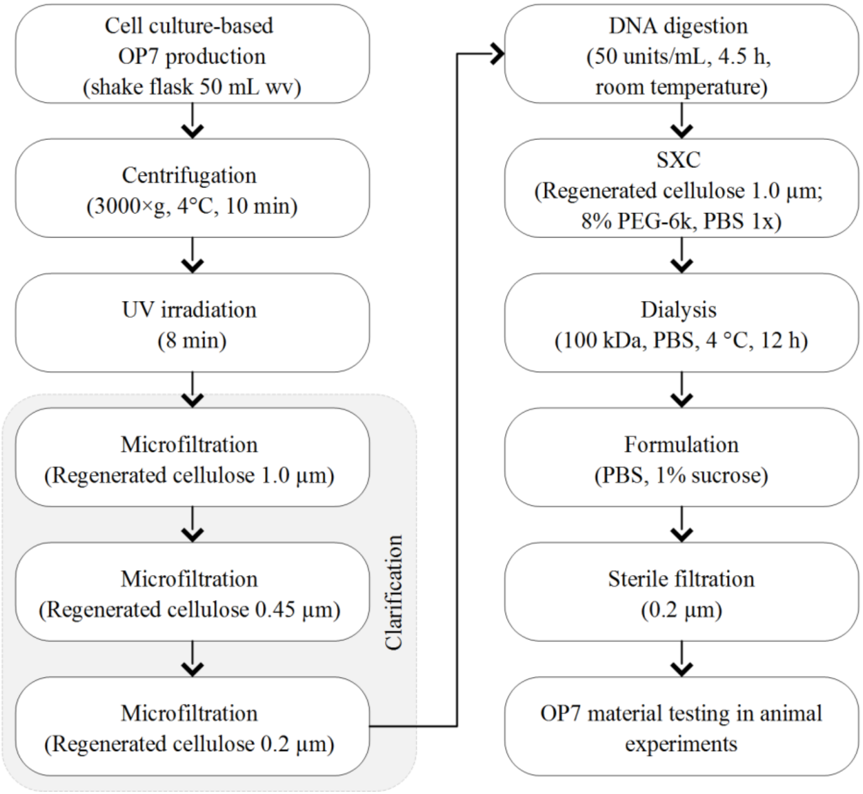


**Fig. S1: Overview of the production and purification workflow for OP7 material.**

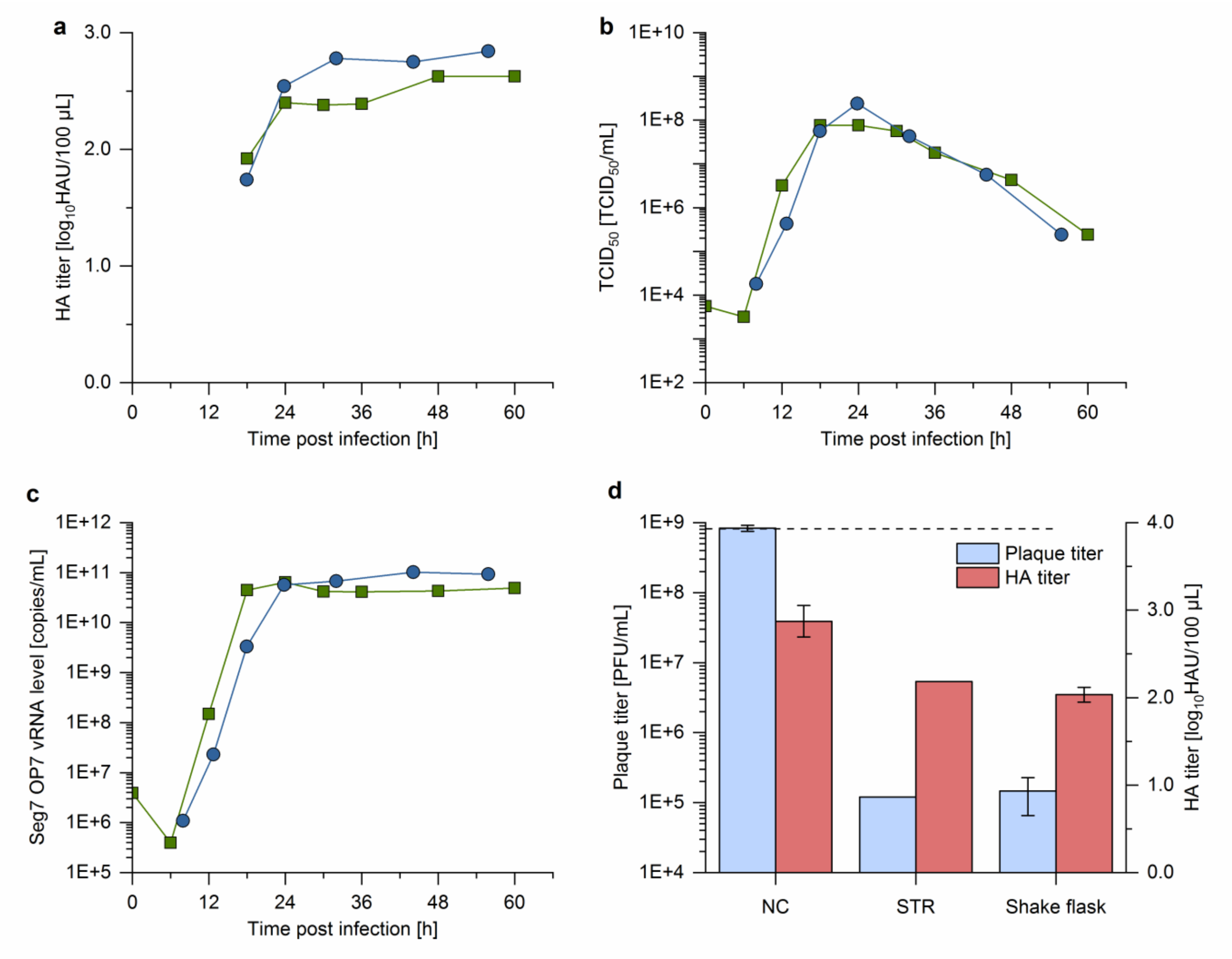


**Fig. S2: Comparison of OP7 production in a stirred tank bioreactor (STR) and a shake flask.** A STR (■) with 500 mL working volume was inoculated with suspension MDCK cells from a shake flask preculture at a VCC of 2E+6 cells/mL. Cells were infected with OP7 seed virus at a MOI 1E-2. For comparison, OP7 production in a shake flask (●) at the same MOI is shown (Fig. 1 and 2). (a) HA titer, (b) $TCID_{50}$ titer, (c) OP7 vRNA level (determined by real-time RT-qPCR), (d) interfering efficacy (see Fig. 2) at optimal harvest time point. Cell culture medium was used as NC for the interference assay. Error bars indicate standard deviation.

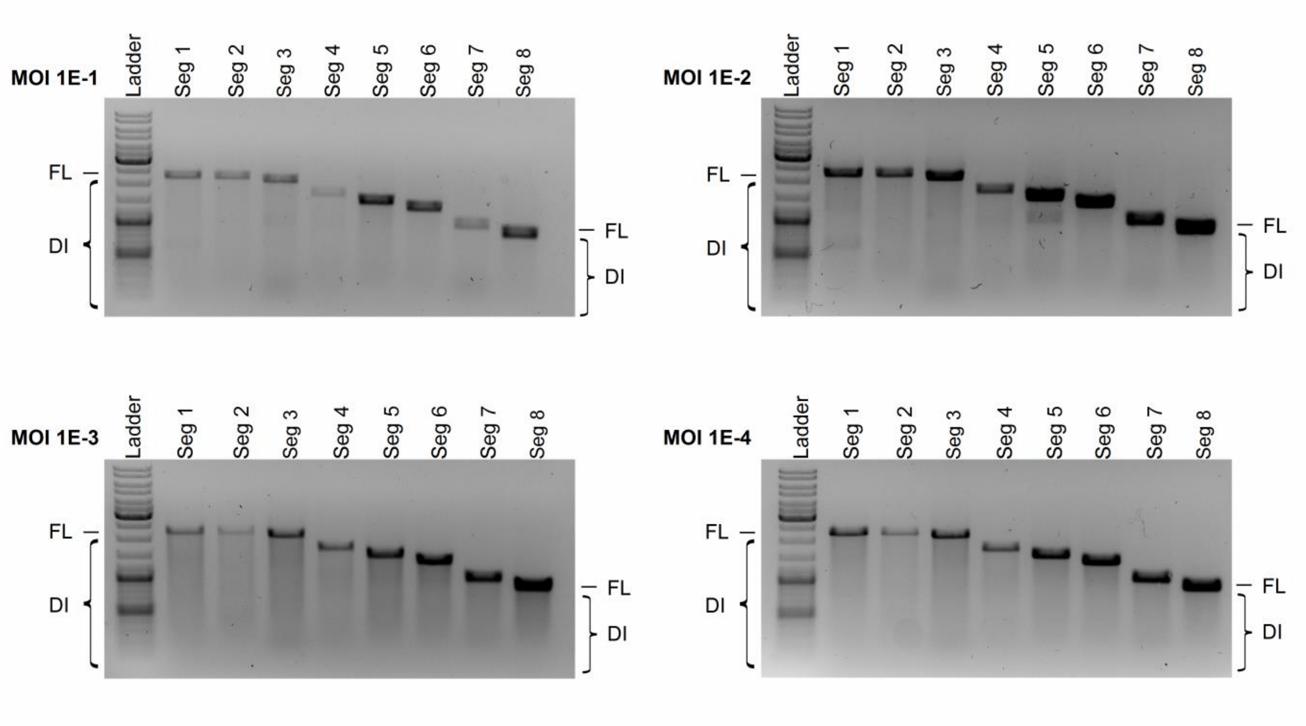


**Fig. S3: Segment-specific RT-PCR of OP7 material produced at different MOIs.** OP7 materials, produced at MOIs ranging from 1E−1 to 1E−4, were tested. Results of all eight genome segments are shown. Signals corresponding to FL and DI vRNAs are indicated. FL size depends on the analyzed vRNA segment. Ladder: upper thick band 3.0 kb, middle thick band 1.0 kb, lower thick band 0.5 kb.

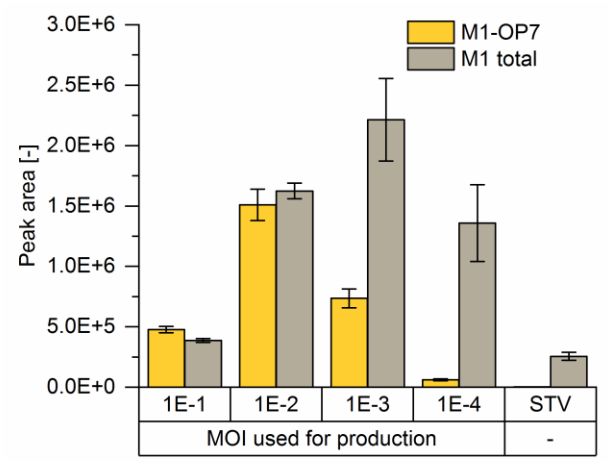


**Fig. S4: MS measurements of M1-OP7 and total M1 of OP7 material produced at different MOIs.** OP7 materials, produced at MOIs ranging from 1E−1 to 1E−4, and as a control, a plaque-purified pure STV, were tested. MS measurements were performed as technical replicates (n=3) of one production sample. Error bars indicate standard deviation.

**Table S1: Tagged primers used for RT**

| **Target** | **Primer name** | **Sequence (5'→3')** |
|---|---|---|
| Seg5 | S5 tagRT for | ATTTAGGTGACACTATAGAAGCGAGTGATTATGAGGGACGGTTGAT |
| Seg7 OP7 | S7-OP7 tagRT for | ATTTAGGTGACACTATAGAAGCGACTGTGACTGCTGAAGTGGTG |
| Seg8 | S8 tagRT for | ATTTAGGTGACACTATAGAAGCGGATAGTGGAGCGGATTCTG |

**Table S2: Primers used for real-time qPCR**

| **Target** | **Primer name** | **Sequence (5'→3')** |
|---|---|---|
| Introduced tag sequence | vRNA tagRealtime for | ATTTAGGTGACACTATAGAAGCG |
| Seg5 | Seg 5 Realtime rev | CGCACTGGGATGTTCTTC |
| Seg7 OP7 | Seg 7 OP7 Realtime rev | CATTTGCCTAGCCCGAATC |
| Seg8 | Seg 8 Realtime rev | CACTTTCTGCTTGGGTATGA |

**Table S3: Virus quantification of different OP7 preparations.**

| OP7 Preparation | Assay | Production replicate 1 | Production replicate 2 | Production replicate 3 (used for animal experiments) |
|---|---|---|---|---|
| **Seed** | HA assay ($log_{10}$ HA units/100 µL) | 1.93 | | |
| | $TCID_{50}$ assay ($TCID_{50}$/mL) | 1.30E+08 | | |
| | Real-time RT-qPCR (Seg7 OP7 vRNA copies/mL) | 5.60E+10 | | |
| **Production MOI 1E−1 (32 hpi)** | HA assay ($log_{10}$ HA units/100 µL) | 2.45 | 2.23 | |
| | $TCID_{50}$ assay ($TCID_{50}$/mL) | 1.00E+07 | 5.6E+05 | |
| | Real-time RT-qPCR (Seg7 OP7 vRNA copies/mL) | 3.19E+09 | 1.09E+10 | |
| **Production MOI 1E−2 (32 hpi)** | HA assay ($log_{10}$ HA units/100 µL) | 2.78 | 2.81 | 2.61 |
| | $TCID_{50}$ assay ($TCID_{50}$/mL) | 4.30E+07 | 7.60E+07 | 5.12E+07 / 0.00 (after UV treatment) |
| | Real-time RT-qPCR (Seg7 OP7 vRNA copies/mL) | 6.62E+10 | 3.43E+10 | 2.20E+10 |
| **Production MOI 1E−3 (32 hpi)** | HA assay ($log_{10}$ HA units/100 µL) | 3.04 | 3.04 | |
| | $TCID_{50}$ assay ($TCID_{50}$/mL) | 5.60E+08 | 4.30E+08 | |
| | Real-time RT-qPCR (Seg7 OP7 vRNA copies/mL) | 7.80E+10 | 1.40E+10 | |
| **Production MOI 1E−4 (32hpi)** | HA assay ($log_{10}$ HA units/100 µL) | 2.96 | 2.74 | |
| | $TCID_{50}$ assay ($TCID_{50}$/mL) | 7.60E+08 | 7.60E+08 | |
| | Real-time RT-qPCR (Seg7 OP7 vRNA copies/mL) | 1.22E+10 | 2.37E+09 | |
| **SXC-purified OP7** | HA assay ($log_{10}$ HA units/100 µL) | 3.67 | | |
| | $TCID_{50}$ assay ($TCID_{50}$/mL) | 0.00 | | |
| | Real-time RT-qPCR (Seg7 OP7 vRNA copies/mL) | 1.12E+11 | | |
